# Supplementary material for: Novel Microsatellite Loci, Cross-Species Validation of Multiplex Assays, and By-Catch Mitochondrial Genomes on Ochthebius Beetles from Supratidal Rockpools
Source: Insects. 2023 Nov 15;14(11):881. doi: 10.3390/insects14110881 (PMC10672297; doi:10.3390/insects14110881)
Supplement: Supplementary file 1 [file insects-14-00881-s001.zip › supplementary_material.pdf]

Supplementary Material

Table S1. Site location information and analysis performed on samples

Locality	Coordinates (LAT; LON)	Species	Method
Denia (Spain)	38.833528; 0.138467	*O. quadricollis*	Testing SSR markers and multiplex optimization
La Illeta (Spain)	38.431639; -0.380675	*O. quadricollis*	Testing SSR markers and multiplex optimization
Santa Pola (Spain)	38.197111; -0.514417	*O. lejolisii*	Testing SSR markers and multiplex optimization
Cala Reona (Spain)	37.617328; -0.712847	*O. quadricollis*	Library sequenced and Mitochondrial genome
		O. subinteger	Testing SSR markers and multiplex optimization
Cala Pulgas (Spain)	37.469876; -1.470352	*O. lejolisii*	Library sequenced and Mitochondrial genome
		O. quadricollis	Testing SSR markers and multiplex optimization
Cala Conchas (Spain)	37.284133; -1.730989	*O. lejolisii*	Testing SSR markers and multiplex optimization
Dwejra Bay (Malta)	36.051210; 14.189570	*O. celatus*	Testing SSR markers and multiplex optimization
Saint Paul's Bay (Malta)	35.949400; 14.401740	*O. celatus*	Testing SSR markers and multiplex optimization

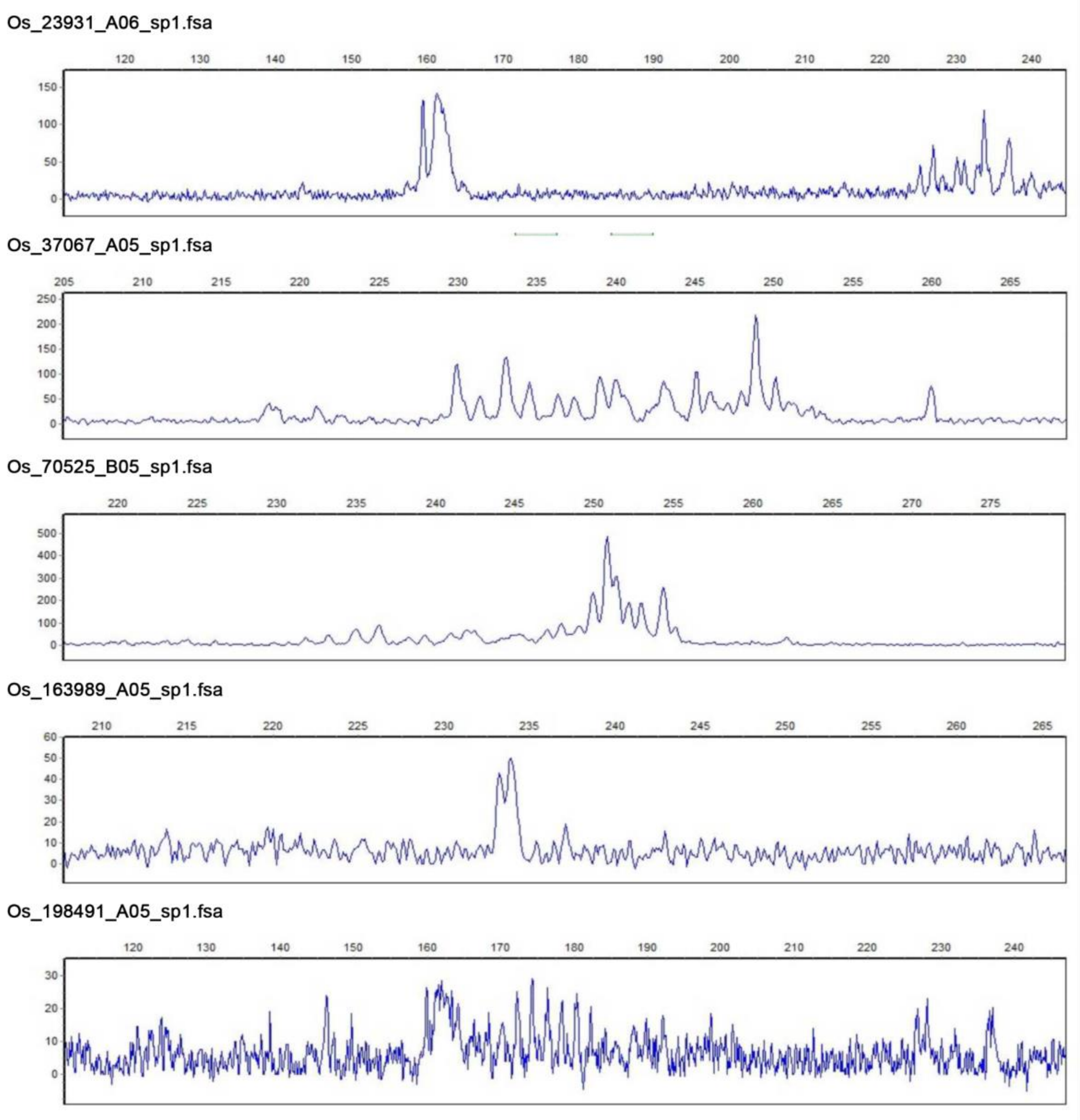

Figure S1. Example of five markers initially designed from the *O. lejolisii* library, where the electropherogram shows unclear amplification for *O. quadricollis* specimens.

Table S2 (Excel format in a separate file). Details and sequences of the loci selected from the *O. lejolisii* and *O. quadricollis* libraries (source of samples in Table S1) for the amplification test.
