# Supplementary figures and images for: Novel Microsatellite Loci, Cross-Species Validation of Multiplex Assays, and By-Catch Mitochondrial Genomes on Ochthebius Beetles from Supratidal Rockpools
Source: Insects. 2023 Nov 15;14(11):881. doi: 10.3390/insects14110881 (PMC10672297; doi:10.3390/insects14110881)

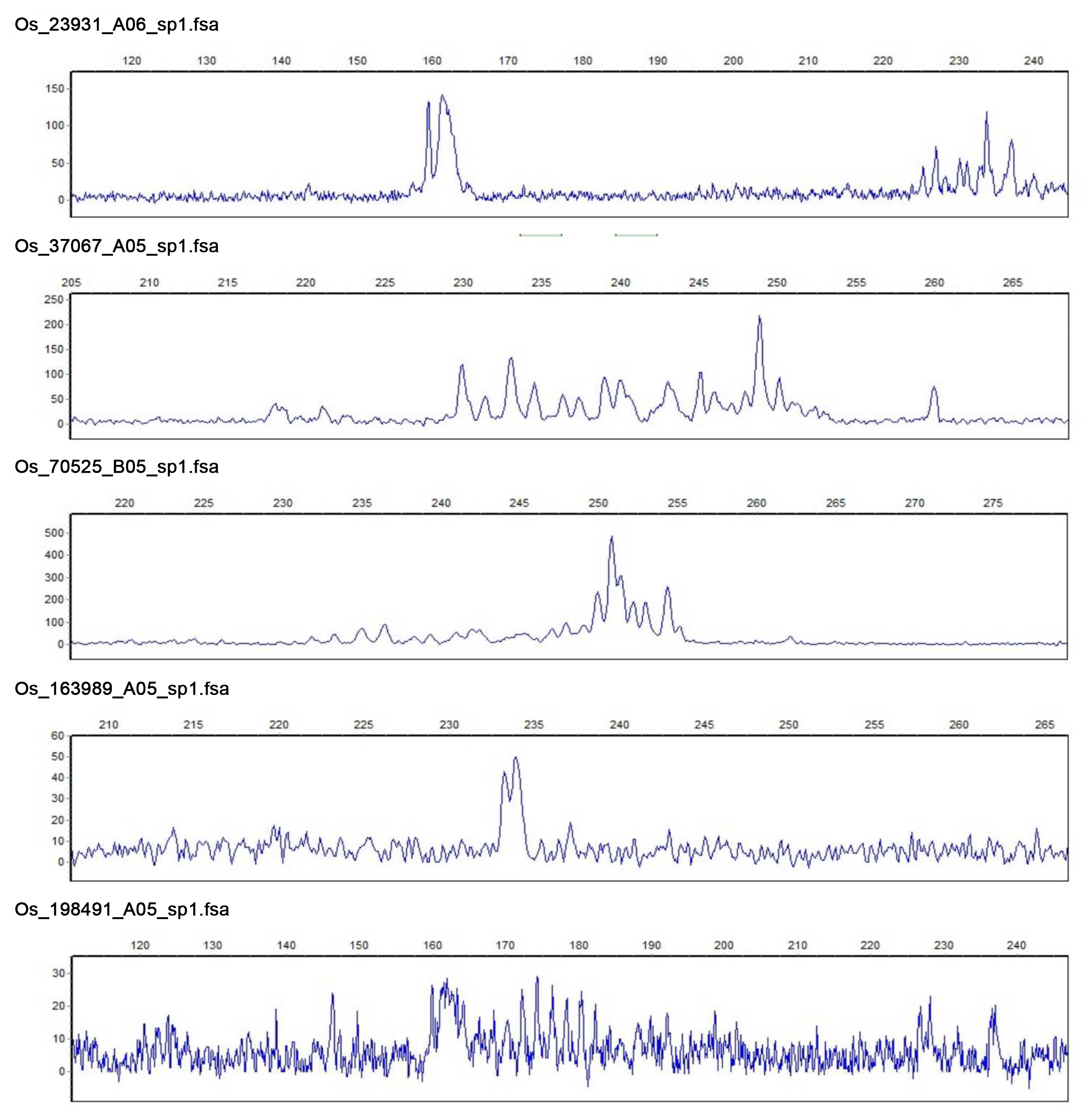

Supplement: Supplementary file 1 [file insects-14-00881-s001.zip › figureS1.tif]
